# The Prevalence of Optical Coherence Tomography Artifacts in High Myopia and its Influence on Glaucoma Diagnosis

**DOI:** 10.1097/IJG.0000000000002268

**Published:** 2023-07-19

**Authors:** Linda Yi-Chieh Poon, Chi-Hsun Wang, Pei-Wen Lin, Pei-Chang Wu

**Affiliations:** * Department of Ophthalmology, Kaohsiung Chang Gung Memorial Hospital, Kaohsiung; † School of Medicine, Chang Gung University, Taoyuan, Taiwan, Republic of China

**Keywords:** artifacts, glaucoma, high myopia, optical coherence tomography, retinal nerve fiber layer

## Abstract

**Précis:**

Optical coherence tomography (OCT) artifacts occur much more frequently in highly myopic eyes compared with non-highly myopic eyes. A longer axial length is predictive of having OCT artifacts.

**Purpose:**

To investigate the types and prevalence of artifacts on OCT scans in patients with and without high myopia.

**Materials and Methods:**

Patients were divided into 4 groups based on whether they had glaucoma and/or high myopia. All peripapillary retinal nerve fiber layer (RNFL) scan images were individually inspected for the presence of artifacts.

**Results:**

Two hundred twenty-six patients were enrolled. The prevalence of OCT artifacts was 18.6% in non-high myopes and 51.9% in high myopes (*P*<0.001). Outer RNFL border misidentification was the most common type of artifact for non-high myopes, whereas retinal pathology-related artifact was the most common in high myopes. Univariable regression analysis showed that a longer axial length [odds ratio (OR) 1.815, *P*<0.001], a higher pattern standard deviation (OR 1.194, *P*<0.001), and thinner RNFL (OR 0.947, *P*<0.001) were predictive factors for the presence of OCT artifacts. The diagnostic capability of global RNFL thickness before and after manual correction of segmentation errors did not differ for both non-high myopes [area under the receiver operating curve 0.915–0.913 (*P*=0.955)] and high myopes [area under the receiver operating curve 0.906–0.917 (*P*=0.806)].

**Conclusion:**

The prevalence of OCT artifacts was the highest in patients with both high myopia and glaucoma. The most common type of OCT artifact is different for non-high myopes and high myopes. Physicians need to be aware of a higher likelihood of OCT artifacts, particularly in those with a longer axial length, worse visual field, and thinner RNFL thickness.

Glaucoma is a progressive optic neuropathy with characteristic atrophy of the optic nerve resulting from the loss of retinal ganglion cells and their axons. Optical coherence tomography (OCT) facilitates structural analysis of the peripapillary retina, optic disc, and macula, and has become an indispensable tool in the diagnosis of glaucoma over the past decades.^1^

However, these machines are not without limitations. OCT artifacts can be present in 19.9–46.3%^2,3^ of RNFL scans, which can lead to erroneous measurements of the retinal nerve fiber layer (RNFL) thickness, misleading clinicians to an incorrect glaucoma diagnosis. Recognition of imaging artifacts is thus critical for accurate interpretation of the OCT examination.

Ocular pathologic features are possible sources of OCT artifacts.^2^ Patients with high myopia are known to have a higher prevalence of peripapillary retinal changes including peripapillary atrophy (PPA) and peripapillary retinoschisis,^4,5^ which could lead to a higher frequency of OCT artifacts occurring in this group of patients.

Past studies that investigated OCT artifacts in peripapillary RNFL scans^2,3^ are limited by a lack of clearly defined refractive status and likely did not include much of highly myopic patients. There was one study^6^ that evaluated the influence of myopia on OCT segmentation errors and glaucoma diagnosis which found a segmentation artifact rate of 44% on 3.45 mm pericapillary RNFL scans. Glaucoma diagnostic capability of RNFL thickness improved from 0.827 to 0.888 [area under the receiver operating curve AUC)] after manual correction of segmentation artifacts; however, the study only included patients with myopia of<−3.0 Diopter (D).^6^ The risk of glaucoma is, however, much higher in patients with high myopia compare with those with mild or moderate myopia.^7,8^ Therefore, it would be important to know how reliably OCT can help clinicians diagnose glaucoma in a group of highly myopic patients.

The purpose of this study is to evaluate the prevalence rate of different types of imaging artifacts on OCT peripapillary RNFL scans in normal patients and in patients with either glaucoma, high myopia, or both. The study also aims to further investigate factors associated with the presence of artifacts and assess how glaucoma diagnosis may be influenced by OCT artifacts.

## MATERIALS AND METHODS

This is a prospective study that consecutively enrolled patients who visited the glaucoma service of Kaohsiung Chang Gung Memorial Hospital between the period of Jan. 2019 to Jan. 2021. The study protocol was approved by the Chang Gung Medical Foundation Institutional Review Board (IRB number 201800926B0) and was conducted in accordance with the tenets of the Declaration of Helsinki. Informed consent was obtained from all of the patients.

All of the patients underwent a comprehensive eye examination that included history, visual acuity, automated refraction, intraocular pressure, axial length measurement (IOLMaster, Carl Zeiss Meditec), visual field (VF) testing (Swedish Interactive Threshold Algorithm standard 30-2 test; Humphrey VF Analyzer 750i, Carl Zeiss Meditec Inc.), slit lamp biomicroscopy, dilated fundus examination, and OCT peripapillary RNFL scan (Spectralis OCT, Heidelberg Engineering). The peripapillary RNFL scan was obtained using a circular scan with a diameter of 12 degrees centered on the optic disc.

Patients were divided into 4 groups based on their refractive status and whether they had glaucoma or not: non-high myope controls, non-high myope glaucoma patients, highly myopic patients, and highly myopic patients with glaucoma. Patients were classified as having high myopia if they had a spherical equivalent (SE) refraction of ≤ – 6.0 Diopter (D) or an axial length of ≥26 mm if the patient had previously undergone cataract surgery or laser refractive surgery. Diagnosis of glaucoma was based on a glaucomatous disc appearance with a typical glaucomatous VF defect (paracentral defect, nasal step, arcuate scotoma, generalized depression, or altitudinal defect) that is repeatable and compatible with the disc appearance. A glaucomatous VF defect was defined as a cluster of 3 or more contiguous test locations on the same side of the horizontal meridian in the pattern standard deviation plot that was depressed at the *P*<0.05 level with at least 1 at the *P*<0.01 level. Only reliable VFs, as defined by a fixation loss of <20%, false-positive rate of <15%, and false-negative rate of <15%, were included in the analysis. Non-high myope controls were those with normal VF, SE of >−6.0 D and <+3.0D, and axial length of <26 mm.

Eyes were excluded from analysis if they had a history of traumatic eye injury, history of vitreoretinal surgery, maculopathy, vascular occlusive retinopathy, proliferative diabetic retinopathy, or non-glaucomatous optic neuropathy, which could result in VF loss not attributable to glaucoma. If both eyes of a patient were eligible for inclusion in the study, one eye was selected randomly using an online randomization tool (https://www.randomlists.com).

**FIGURE 1:**
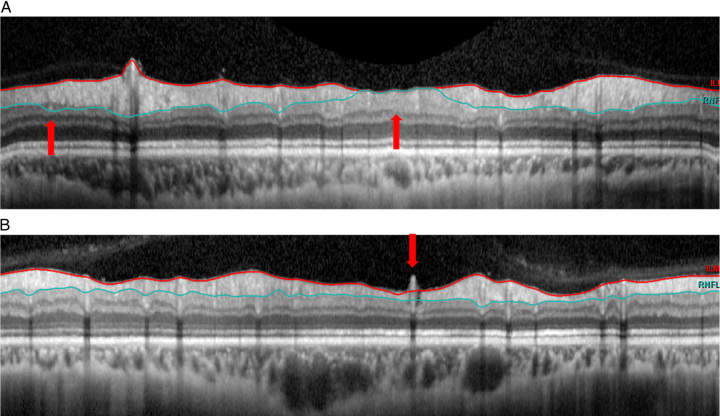
Examples of artifacts categorized under software algorithm failure. A, Outer retinal nerve fiber layer (RNFL) border misidentification (thick red arrow). B, Inner RNFL border misidentification (thick red arrow).

**FIGURE 2:**
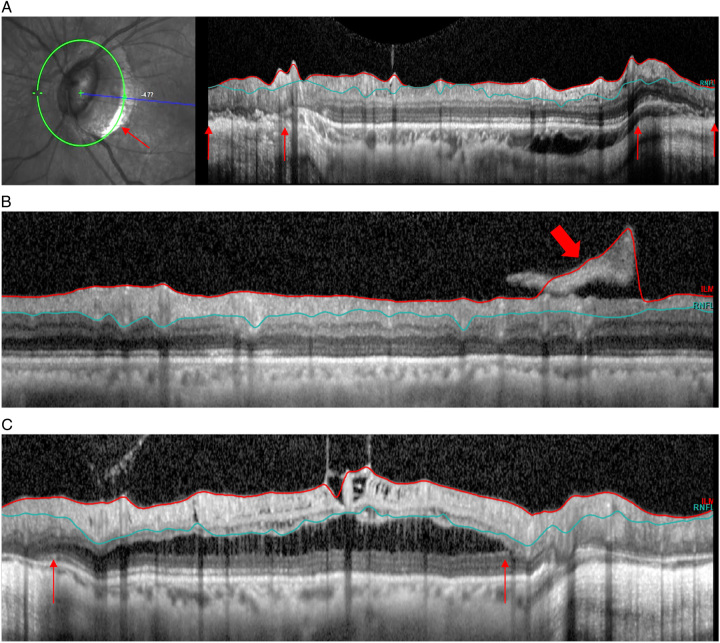
Examples of artifacts categorized under retinal pathology pathology-related artifacts. A, Peripapillary atrophy (in between thin red arrows). B, Posterior vitreous detachment/epiretinal membrane (thick red arrow). C, Peripapillary retinoschisis (in between thin red arrows).

**FIGURE 3:**
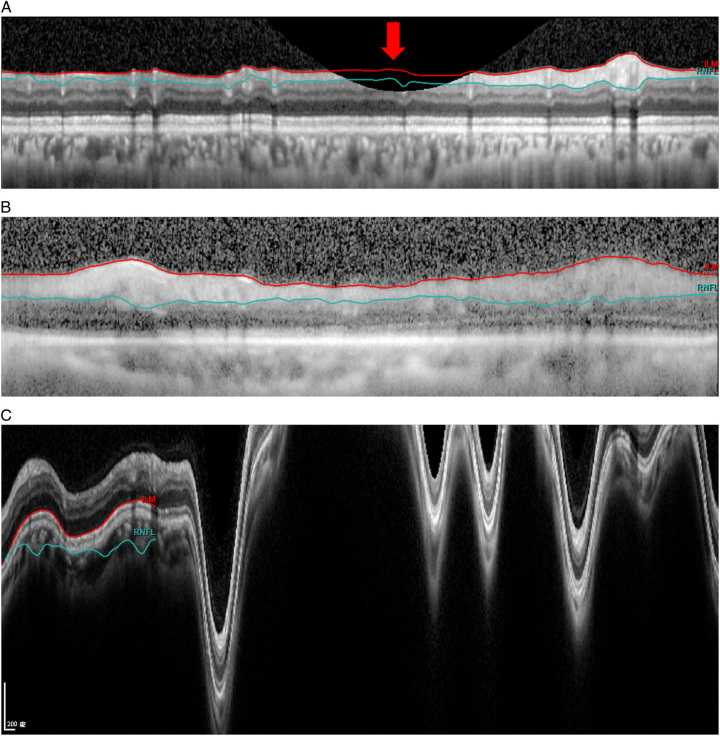
Examples of artifacts categorized under image acquisition artifacts. A, Truncated image (thick red arrow). B, Low signal. C, Motion artifact.

All of the patients’ peripapillary RNFL scan images were reviewed by 2 experienced examiners (L.Y.-C.P., C.-H.W.) to assess for the presence of image artifacts. The prevalence and types of artifacts were recorded for analysis. With modifications based on the classifications used by past studies,^2,3,9^ we divided OCT image artifacts on RNFL scans into 3 categories: software algorithm failure, retinal pathology-related artifacts, and image acquisition artifacts. Under the category of software algorithm failure, 3 types of OCT artifacts were identified, and included inner RNFL border misidentification, outer RNFL border misidentification (Fig. 1), and complete segmentation failure. The category of retinal pathology-related artifacts included artifacts due to the presence of PPA, peripapillary retinoschisis, and posterior vitreous detachment and/or epiretinal membrane (PVD/ERM) (Fig. 2). Truncated image, low signal, and motion artifacts fall under the category of image acquisition artifacts (Fig. 3). Definitions of each type of artifact are summarized in Table 1. Only segmentation errors that occurred in the absence of retinal pathology or acquisition error are considered segmentation algorithm failure. Segmentation errors that occurred due to the presence of retinal pathology or acquisition error are counted as only the retinal pathology or acquisition error itself. However, more than 1 type of OCT artifact may be present on a single RNFL scan such as having both motion artifact and truncation artifact or having both PVD/ERM and PPA.

**TABLE 1:** Definition of the Optical Coherence Tomography Artifacts Examined in this Study

Type of artifacts	Definition
Software algorithm failure	
Inner RNFL border misidentification	Incorrect segmentation of the inner RNFL border
Outer RNFL border misidentification	Incorrect segmentation of the outer RNFL border
Complete segmentation failure	No segmentation line along the RNFL borders
Retinal pathology-related artifacts	
PPA	Incorrect segmentation of the RNFL borders due to the scan circle overlapping peripapillary atrophy
PVD/ERM	Misidentification of the inner RNFL border due to the presence of posterior vitreous detachment or epiretinal membrane
Peripapillary retinoschisis	Incorrect segmentation of the RNFL borders due to the presence of retinoschisis
Image acquisition artifacts	
Truncated image	Vertically displaced image resulting in part of the inner or outer retina not within the acquisition window of the scan
Low signal	Retinal layers indistinguishable from the background noise along a portion or the entire length of the image
Motion artifact	Wavy or undulating appearance of the scan image due to movement during the scan resulting in indiscernible retinal layers

PPA indicates peripapillary atrophy; PVD/ERM, posterior vitreous detachment/epiretinal membrane; RNFL, retinal nerve fiber layer.

In those with OCT artifacts resulting in segmentation errors, manual correction of the segmentation error was performed to assess the influence of OCT artifacts on RNFL thickness measurements and its glaucoma diagnostic capability.

Statistical analyses were performed using the SPSS Statistics software, version 26 (IBM Corp.). For continuous variables that are normally distributed, comparison across groups was performed using the one-way ANOVA, and are expressed as mean±SD. Parameters that are not normally distributed are expressed as median (interquartile range) and are compared using the Kruskal-Wallis test. Comparisons of the artifact prevalence rate were performed using the χ^2^ test. Logistic regression analysis was used to determine factors associated with the occurrence of OCT artifacts. The variance inflation factor was used to assess for multicollinearity between variables used for the regression analysis model. The glaucoma diagnostic capability of OCT RNFL thickness was assessed by using the AUC analysis. Comparison of AUC value between non-high myopes and high myopes is performed using MedCalc Statistical software version 20.012 (MedCalc Software Ltd). A *P* value of ˂0.05 was considered statistically significant.

## RESULTS

Two hundred twenty-six scans of 226 patients were enrolled in the study, with the patients having an average age of 49.0±13.5 years. The study included 61 non-high myope control, 57 non-high myope glaucoma patients, 62 high myopes without glaucoma, and 46 high myopes with glaucoma. The baseline demographics of the study population are summarized in Table 2. Eighteen (8.0%) eyes were pseudophakic and 17 (7.5%) eyes had previously undergone laser refractive surgery. The spherical equivalent of the non-highly myopic patients was −2.56±2.16 D, and −9.71±3.14 D in the highly myopic patients.

**TABLE 2:** Baseline Demographics of the Study Population

	Normal (n=61)	Glaucoma (n=57)	High myopia without glaucoma (n=62)	High myopia with glaucoma (n=46)	*P*
Age^*^ (y)	48.0 (33.0–56.5)	61.0 (54.0–69.0)	43 (32.0–49.0)	53 (42.8–57.0)	<0.001
IOP^*^ (mmHg)	15.7±3.2	13.3±3.0	15.6±3.0	14.2±3.5	<0.001
SE^*^ (D)	−2.45±2.12	−2.69±2.21	−9.67±3.10	−9.80±3.26	<0.001
AL^*^ (mm)	24.93±1.11	25.19±1.21	27.53±1.28	27.74±1.42	<0.001
VF MD^*^ (dB)	−0.29 (−1.64 to 0.82)	−2.93 (−9.29 to −0.92)	−1.44 (−2.56 to −0.10)	−6.04 (−13.87 to −2.55)	<0.001
VF PSD^*^ (dB)	1.82 (1.54–2.13)	4.59 (2.39–10.36)	2.14 (1.61–2.94)	7.07 (3.33–13.27)	<0.001
RNFLT- global^*^ (microns)	97.51±10.32	72.63±14.92	87.07±13.02	63.43±13.12	<0.001
Prior surgery					
Cataract surgery, n (%)	0	6 (10.5)	2 (3.2)	10 (21.7)	<0.001
Laser refractive surgery, n (%)	4 (6.6)	0	4 (6.5)	9 (19.6)	<0.001

* Values are presented as mean±SD or as median (interquartile range).

AL indicates axial length; D, diopter; dB, decibel; IOP, intraocular pressure; RNFLT, retinal nerve fiber layer thickness; SE, spherical equivalent; VF MD, visual field mean deviation; VF PSD, visual field pattern standard deviation.

For the entire population, OCT artifact was present in 78 out of 226 RNFL scans (34.5%). Out of the 78 scans with OCT artifacts, 71.8% (56 out of 78) of patients were highly myopic, whereas 28.2% were non-highly myopic (*P*<0.001). The most common category of OCT artifacts for the entire population was retinal pathology-related artifact, which was present in 39 out of 226 (17.3%) scans. Segmentation algorithm failure occurred in 15.9% (36 out of 226) of scans. Image acquisition artifact was the least common and occurred in 13 out of 226 (5.8%) scans.

**FIGURE 4:**
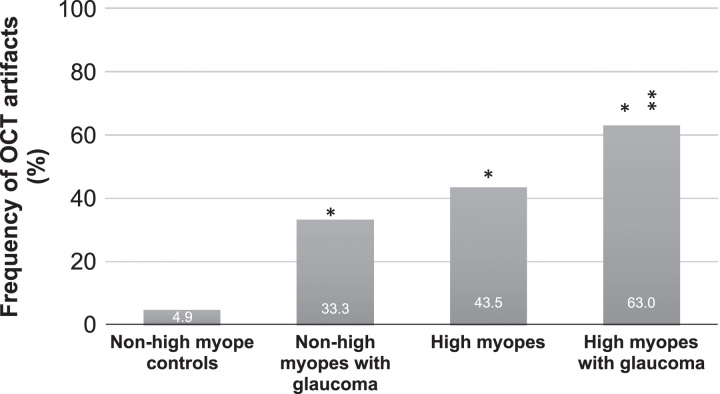
The prevalence of optical coherence tomography (OCT) artifact in non-high myope controls, non-high myopes with glaucoma, high myopes without glaucoma, and high myopes with glaucoma *represents statistically significant difference compared to non-high myope controls. **represent statistically significant difference compared to non-high myopes with glaucoma).

When divided into the non-high myope and high myope groups, the prevalence rate of OCT artifact was found to be 18.6% in non-high myopes and 51.9% in high myopes (*P*<0.001). Table 3 shows the prevalence rate for all of the artifacts analyzed in this study. The most common category of OCT artifact is different for non-high myopes and high myopes. In highly myopic patients, the most common category of OCT artifact is retinal pathology-related artifacts with PPA as the most frequent cause of OCT artifacts (25 out of 108 scans, 23.1%). In the non-high myopes, the most common category of OCT artifact is software algorithm failure with outer RNFL border misidentification as the most frequent type of artifact (13 out of 118 scans, 11.0%), and occurred predominantly in glaucoma patients (Table 3). Motion artifacts occurred more frequently in high myopes compared with non-high myopes [10 out of 108 scans (9.3%) vs. 1 out of 118 scans (0.8%), respectively, *P*=0.004]. Relatively low and similar prevalence rates between high myopes and non-high myopes were found for PVD/ERM, peripapillary retinoschisis, truncated image, and low signal artifacts (Table 3). Overall, OCT artifact is relatively uncommon in a non-highly myopic normal population (4.9%, Fig. 4); however, the prevalence of OCT artifacts becomes much higher with a patient having glaucoma (33.3%), high myopia (43.5%), or both (63.0%) (Fig. 4).

**TABLE 3:** Frequency of Each Type of Artifact in the Study Population

	Normal (n=61)	Non-high myopes with glaucoma (n=57)	High myopes without glaucoma (n=62)	High myopes with glaucoma (n=46)	*P*
Software algorithm failure					
Outer RNFL border misidentification, n (%)	2 (3.3)	11 (19.3)	10 (16.1)	11 (23.9)	0.016
Inner RNFL border misidentification, n (%)	0	0	1 (1.6)	2 (4.3)	0.185
No segmentation, n (%)	0	0	0	0	1.000
Retinal pathology-related artifact					
PPA, n (%)	0	4 (7)	15 (24.2)	10 (21.7)	<0.001
PVD/ERM, n (%)	1 (1.6)	3 (5.3)	2 (3.2)	4 (8.7)	0.333
Peripapillary retinoschisis, n (%)	0	1 (1.8)	3 (4.8)	2 (4.3)	0.321
Image acquisition artifact					
Truncated image, n (%)	0	2 (3.5)	1 (1.6)	0	0.314
Low signal, n (%)	0	1 (1.8)	1 (1.6)	0	0.610
Motion artifact, n (%)	0	1 (1.8)	5 (8.1)	5 (10.9)	0.026

PPA indicates peripapillary atrophy; PVD/ERM, posterior vitreous detachment/epiretinal membrane; RNFL, retinal nerve fiber layer.

To identify predictive factors associated with the presence of OCT artifacts on RNFL scans, logistic regression analysis was performed. The results of the regression analysis are summarized in Table 4. Univariable regression analysis found that axial length, VF pattern standard deviation, global RNFL thickness, and RNFL thickness in the superotemporal and inferotemporal sectors were significant predictive factors associated with having artifacts on OCT RNFL scans. Age, IOP, and scan quality score were not associated with the presence of OCT artifacts. In the multivariable model, only axial length [odds ratio 1.837, 95% CI (1.430–2.360), *P*<0.001] remained significantly associated with the presence of OCT artifact.

**TABLE 4:** Univariable Regression Analysis for the Predictors of the Presence of any Optical Coherence Tomography Artifacts

Variables	Coefficient	OR (95% CI)	*P*
Age	0.017	1.017 (0.996–1.038)	0.115
IOP	−0.082	0.921 (0.845–1.005)	0.065
Axial length	0.596	1.815 (1.479–2.227)	<0.001
VF PSD	0.177	1.194 (1.114–1.279)	<0.001
Scan Quality score (Q)	−0.055	0.946 (0.876–1.023)	0.164
Retinal nerve fiber layer thickness			
Global	−0.045	0.947 (0.929–0.965)	<0.001
Superonasal sector	0.001	1.000 (0.996–1.004)	0.985
Superotemporal sector	−0.013	0.987 (0.979–0.996)	0.003
Temporal sector	−0.005	0.995 (0.985–1.005)	0.347
Inferotemporal sector	−0.018	0.982 (0.975–0.989)	<0.001
Inferonasal sector	−0.002	0.998 (0.990–1.005)	0.543
Nasal sector	0.001	1.001 (0.998–1.005)	0.398

IOP indicates intraocular pressure; OR, odds ratio; VF PSD, visual field pattern standard deviation.

In OCT scans that had artifacts (78 out of 226 scans), segmentation errors were manually corrected to assess the influence of OCT artifacts on RNFL measurements and their diagnostic capability. After inspection and correction of the segmentation errors, it was found that 7 out of 78 scans (8.9%) could not be segmented, all of which contained motion artifacts and precluded the generation of RNFL thickness data and were not included in the calculation of AUC value. Despite manual correction of the segmentation errors, the diagnostic capability of global RNFL thickness in non-high myopes remained relatively unchanged from an AUC value of 0.915–0.913 (*P*=0.955). In high myopes, the AUC increased slightly from 0.906 to 0.917, but the difference was also not statistically significant (*P*=0.806).

## DISCUSSION

Although several OCT parameters have been proposed and investigated for glaucoma diagnosis,^10^ RNFL thickness remains one of the most commonly used and reported parameters in OCT for monitoring and detection of glaucoma;^1^ however, OCT-based diagnosis for glaucoma using RNFL thickness relies considerably on the accuracy of the RNFL measurements, which can be significantly influenced by the presence of OCT artifacts.^6,11^ Although it was often assumed in the past studies that high myopia can be a cause of OCT artifacts on RNFL scans,^2,3,6^ the past studies have not clearly demonstrated this relationship. In this study, we comprehensively analyzed the frequency of artifacts on RNFL scans of the Spectralis OCT in a population that included non-high myope controls, non-high myope glaucoma patients, highly myopic patients, and highly myopic patients with glaucoma. Our study showed that increased axial length is a predictive factor for the presence of OCT artifacts and we also found that OCT artifacts were present in over 50% of highly myopic patients, with the prevalence increasing to over 60% if the highly myopic patient also has glaucoma.

In our study, we found that outer RNFL border misidentification was the most common type of OCT artifact in non-high myopes and occurred predominantly in glaucoma patients (Table 3). On OCT, the adjacent layers of the retina are visualized as different scattering intensities or reflectances.^12^ Segmentation algorithms are designed to detect and delineate these intraretinal boundaries; however, reduced contrast between the adjacent retinal layers can limit the ability of the algorithm to accurately detect the boundaries of interest.^13^ The RNFL can be observed as a highly reflective layer of the retina with the internal limiting membrane as its inner border and the boundary between the RNFL and the more hyporeflective retinal ganglion cell (RGC) layer as its outer border. However, the reflectivity of the RNFL decreases with the progression of glaucoma,^14–16^ which can make the border between the RNFL and the RGC layer less distinguishable, resulting in outer RNFL border misidentification. The association of glaucoma severity with the presence of OCT artifacts is also supported by the finding in previous studies.^3,17^ The loss of RNFL reflectivity in glaucomatous disease may in part be explained by the changes in cytoskeletal components of the RGC axons and their supporting cells with the progression of the disease.^18–20^

In high myopes, outer RNFL border misidentification was also common and occurred in 21 out of 108 (19.4%) scans of highly myopic patients. Although there have been no prior studies that investigated the differences in RNFL reflectivity in myopic eyes, there was one study that demonstrated changes in the cytoskeletal architecture and thinning of the RGC axons in chicken eyes with induced myopia,^21^ and by inference, could also have an impact on the reflectivity of the RNFL.^18^ In addition, myopia is known to cause axial elongation and deformation of the eyeball, which has previously been well demonstrated on 3-dimensional magnetic resonance imaging.^22,23^ Extreme elongation of the globe may result in an inadequately captured image due to exceeding the diopter compensation of the device or from insufficiently reflected image signals.^24^ Distortion of the posterior pole may also result in a highly curved peripapillary retinal image on OCT,^6^ and could hinder the segmentation algorithm from identifying the outer border of the RNFL accurately. Furthermore, irregularity in the contour of the peripapillary region can lead to a variable focusing effect and irregular light scattering resulting in images with insufficient clarity and subsequently leading to a higher likelihood of segmentation errors.

Retinal pathology-related artifact, which includes OCT artifacts that occurred due to the presence of PPA, PVD/ERM, and/or peripapillary retinoschisis, was the most common category of artifact in our highly myopic patients. In contrast to the findings by Asrani et al^2^ who found that ERM was the most common ocular pathology resulting in OCT artifact, we found that PPA was the most frequent cause of retinal pathology-related artifacts (Table 3). Our study population, however, included mostly myopic patients, whereas the refractive status of the patient population by Asrani et al^2^ was not specified. PPA is very common in highly myopic patients and can be present in as many as 79%–100% of highly myopic eyes.^5,25^ We regarded PPA as a cause of OCT artifact only if it was overlapped by the OCT scan circle and found a significantly higher prevalence of PPA-associated artifact in high myopes (23.1%) compared with non-high myopes (4%) (*P*<0.001). This suggests that not only is PPA more common in high myopes but also PPAs larger than the 12 degrees (3.4–3.5 mm diameter) scan circle are more common in high myopes. On OCT, PPA can appear as the loss or disruption of the RPE layer, RNFL plaques, retinal thinning, and abnormal retinal sloping.^26^ Such loss of the normal retinal architecture or layers may subsequently result in the inability of the segmentation algorithm to accurately detect the RNFL borders.^3,17,27^ Although peripapillary retinoschisis is also considered a characteristic finding in highly myopic patients,^4,5^ we found the occurrence of retinoschisis to be low and affected 5 out of 108 scans (4.6%) in highly myopic patients.

Image acquisition artifacts, which included truncated image, low signal, and motion artifacts, are considered technician-dependent,^2,3,27^ and could be decreased with adequate technician training and experience.^27^ In this study, we found a relatively low prevalence of ˂1% for truncated image and low signal artifacts in both non-high myope and high myopes, which suggests that our technicians have been adequately trained to avoid these types of technician-dependent artifacts; however, a significantly higher prevalence of 9.3% for motion artifact, compared with a 0.8% prevalence in non-high myopes, was still noted in high myopes (*P*=0.004) and could reflect the difficulties in reaching an adequate focal plane, needing frequent adjustments to obtain an OCT image in high myopes as their eyeballs are more elongated than a typical normal eye.^28,29^

Although often assumed to be associated with the occurrence of OCT artifacts, none of the past studies have clearly demonstrated the relationship between high myopia and OCT artifacts.^2,6^ The result of our multivariable regression analysis showed that the likelihood for the presence of OCT artifact increased by 1.8 times for every 1 mm increase in axial length, which verifies myopia as a risk factor for having OCT artifacts. In our univariable regression analysis, we also found that increased glaucoma severity and thinner RNFL thickness are associated with the presence of OCT artifacts, which is in agreement with previous studies.^3,6,17^ Although age was previously reported by Li et al^17^ to be associated with the occurrence of OCT artifacts, we did not find age to be a predictive factor in either the univariate or multivariate analysis. Older patients have a higher likelihood of having cataracts, dry eyes, and smaller pupils which may increase the difficulties of obtaining a clear image on OCT; however, our study population is a relatively young population with a mean age of 49 years, which is much younger than the study population in Li et al^17^’s study with a mean age of 72, and could partially explain why age was not found to be a contributory factor for the presence of OCT artifacts in this study.

Although not necessarily resulting in segmentation errors, temporal displacement of the RNFL, torsional changes in the RNFL topography, tilting of the optic disc, and difficulties in determining the center of a myopic disc with incorrect centration of the measurement circle can result in a false classification by the machine of being outside of normal limits (Red disease), which can also confound clinical interpretation.^30^ Furthermore, a magnification effect associated with myopia may result in a thinner measured RNFL thickness,^31^ with some authors advocating adjustment for ocular magnification effect in patients with myopia when making glaucoma diagnosis.^31^

Although studies in the past have demonstrated a good to excellent glaucoma diagnostic capability of OCT RNFL thickness in high myopia,^32–36^ not all studies agreed on whether this differs between high myopes or non-high myopes. Although some studies show that the diagnostic capability of OCT was significantly worse in highly myopic subjects,^32^ other studies show that there was no difference between highly myopic versus non-highly myopic subjects.^33,34^ Our study found that despite a prevalence of 51.9% in high myopes and 18.6% in non-high myopes for OCT artifacts, the AUCs for global RNFL thickness was over 0.9 in both groups of patients, suggesting excellent glaucoma diagnostic capability for this OCT parameter. The high AUC value for the RNFL thickness parameter in the highly myopic patients in this study could be the result of the analysis being performed between highly myopic patients with glaucoma to highly myopic normal patients instead of being compared with normal patients without high myopia and suggests the importance of using a myopic normative database when assessing this group of patients. Biswas et al^37^ have also previously demonstrated the importance of incorporating a myopic normative database for RNFL thickness analysis in highly myopic patients by showing a significantly higher diagnostic performance and specificity for glaucoma detection in this group of patients when compared with the machine’s built-in normative database.

We also found that the AUC values did not differ before and after the manual correction of segmentation errors [non-high myopes: AUC 0.915–0.913 (*P*=0.955); high myopes: AUC 0.906 to 0.917 (*P*=0.806)]. Although it is important to correct ambiguous or erroneous segmentation and modify corresponding thickness values before making clinical assessments, manual corrections are time-consuming and may not be feasible in busy clinical settings. The high AUCs before and after the correction of segmentation artifacts suggest that in general, OCT RNFL thickness remains a useful tool for the detection of glaucoma even without the manual correction of segmentation errors. However, it should be acknowledged that during manual correction of segmentation errors, it was found that 7 out of 78 scans with OCT artifacts could not be re-segmented and were not included in the AUC analysis for RNFL thickness, and the high AUC values shown in this study would thus need to be interpreted with caution. Motion artifact was found to be the cause for all of the scans that could not be re-segmented. Therefore, it is important to repeat the RNFL scan when a motion artifact is present, as segmentation is often not feasible when such images are obtained.

In addition, the high AUC for RNFL thickness in the high myopes shown in this study could have resulted from the lower-than-expected prevalence of PPA affecting the RNFL segmentation. PPA is very common in glaucoma and myopic patients, with its prevalence ranging from 68% to 95%^38,39^ and 100%,^5,40^ respectively; however, in this study, we found a prevalence of PPA-related artifact of 7% in non-highly myopic glaucoma patients, and 22%–24% in highly myopic patients. Given the high prevalence of PPA in glaucoma and myopic patients, this finding can be surprising, however; past studies have found a mean maximum radial width for PPA (the distance from the optic disc border to the edge of PPA) of 0.24–0.37 mm,^40–42^ which suggests that in most patients with PPA, the PPA is not large enough to be intersected by the 3.45 mm diameter RNFL scan circle. However, adjustment of the RNFL scan circle diameter to 4.1 and 4.7 mm using the Glaucoma Module Premium Edition software of the Spectralis OCT can be helpful in cases with larger PPAs that are overlapped by the 3.45 mm scans.^43^

Assessment of the neuroretinal rim thickness could be an alternative to measuring peripapillary RNFL thickness in myopic eyes. Previous studies have analyzed the Bruch’s membrane opening—minimum rim width parameter and reported comparable sensitivity and higher specificity for glaucoma diagnosis in myopic eyes compared with the RNFL thickness parameter.^44,45^ However, as the presence of gamma-PPA (peripapillary sclera without overlying retinal layers, choroid, and Bruch’s membrane) is more common in myopic eyes,^46^ and has previously been demonstrated that around 30% of highly myopic eyes have indiscernible Bruch’s membrane opening in at least 1 meridian,^47^ it should be cautioned that the assessment of the neuroretinal rim may also be confounded by myopia.

Macular ganglion cell analysis is another useful adjunct to peripapillary RNFL thickness measurement for evaluating patients with concomitant glaucoma and high myopia. Previous investigations have shown that the macular ganglion cell complex analysis had comparable^33,35,36^ or better^32,48^ glaucoma diagnostic capability than the peripapillary RNFL measurements in highly myopic patients. Similar to the peripapillary RNFL thickness, the implementation of a myopic normative database has been suggested,^36^ which has been shown to improve the diagnostic ability of the macular parameters.^49^

The high prevalence of OCT artifacts on RNFL scans particularly in patients with high myopia should caution clinicians to carefully inspect RNFL segmentation and OCT images individually. Previously, Li et al,^17^ found that in all patients with OCT artifacts, 23.9% of artifacts masked actual RNFL thinning progression and led to a false-negative diagnosis as no glaucoma progression, whereas 36.5% of artifacts led to a false-positive interpretation of RNFL thinning. These findings highlight the possibility of a false glaucoma diagnosis through an erroneous RNFL thickness measurement and the importance of inspecting individual OCT images for artifacts.

Our study has some limitations. Firstly, OCT machines from different manufacturers have their own segmentation algorithm and may use different scanning protocols to generate RNFL thickness.^50^ Therefore the results presented in this study may not be generalizable to OCT images obtained from machines other than the one used in this study (Spectralis OCT, Heidelberg Engineering). Secondly, patients with glaucoma in our study were, in general, older than those without (Table 2), and as older age has previously been found to be associated with the presence of artifacts,^11,17^ this could potentially contribute to a higher prevalence of OCT artifacts in glaucoma patients noted in this study. However, age was not identified as a contributing factor to the presence of OCT artifacts in either the multivariate or univariate regression analysis in this study.

In conclusion, our study found a high prevalence of over 50% for OCT artifacts in highly myopic patients, with the prevalence increasing to over 60% if the highly myopic patient also has glaucoma. The most common type of OCT artifact is outer RNFL border misidentification in non-high myopes and retinal pathology-related artifacts in high myopes. Physicians need to be aware of a higher likelihood of OCT artifacts occurring particularly in those with a longer axial length, worse visual field, and thinner RNFL thickness which can limit the usefulness of OCT in individual cases.
